# It takes two hearts to cope with an artificial one: the necessity of applying a dyadic approach in the context of left ventricular assist device transplantation—Opinion paper

**DOI:** 10.3389/fpsyg.2023.1215917

**Published:** 2023-07-27

**Authors:** Maya Golan, Noa Vilchinsky

**Affiliations:** ^1^The Psycho-Cardiology Research Lab, Department of Psychology, Faculty of Social Sciences, Bar-Ilan University, Ramat Gan, Israel; ^2^Afeka Academic College of Engineering, Tel Aviv-Yafo, Israel

**Keywords:** left ventricular assist device (LVAD), dyadic coping, dyadic interview, chronic illness management, Psycho-Cardiology

“*I am not coping for myself, I am coping for her, so that she will have me for more time” (a quote from a patient coping with LVAD, April, 2022)*.

## Introduction

Heart failure (HF) is a prevalent mortality factor worldwide. According to estimations, 1 in 33 individuals in the U.S. will be coping with HF by 2030 (Savarese and Lund, 2017). In the case of advanced HF, some patients are offered transplantation of a left ventricular assist device (LVAD), which is a mechanical device that replaces the failing heart. Patients coping with LVAD need extensive support, such as help in bathing (because the device must stay dry), in changing sterile bandages around a hole in the abdomen through which the device connects to the heart, and stringent follow-up with medications (Abshire et al., 2016). These tasks are usually attended to by their spouses/cohabitating partners. Thus, we argue that in order to grasp the in-depth meaning of coping in this context, LVAD should be viewed as a dyadic experience and an individual approach is not only insufficient but might even be misleading. This argument is based on our studies in the Psycho-Cardiology Research Lab, Bar-Ilan University, Israel, which focus on couples rather than individual patients coping with different cardiac contexts, LVAD in particular, demonstrating how the dyadic perspective enriches our understanding of patients' coping.

## It takes two to develop negative clinical outcomes

Adopting a dyadic point of view reveals the complexity of the relation between partners' caregiving and coping styles and both partners' clinical outcomes. For example, a compulsive caregiving style (i.e., care provision which is too intrusive; Bowlby, 1982) was found to positively associate with patients' anxiety symptoms (George-Levi et al., 2016), and to moderate the association between patients' anxious attachment orientation and anxiety symptoms (George-Levi et al., 2020). Patients whose partners engaged in a protective buffering coping style (i.e., tendency to deny and to hide worries from the patient; Coyne and Smith, 1994), presented higher depression levels but only when perceiving their partners' buffering coping style as low, thus not protective (Vilchinsky et al., 2011). In a recent publication, we also detected that patients' perceived partners' protective buffering was associated with their cardiac-disease-induced posttraumatic stress symptoms (CDI-PTSS) over time (George-Levi et al., 2022). Interestingly, patients' CDI-PTSS was associated with distress and fear of illness progression among their partners (Eisenberg et al., 2022); and cardiac patients' partners' depression was found to associate with lower marital satisfaction of both patient and partner (Dekel et al., 2013).

## It takes two to engage in health-promoting behaviors

The dyadic point of view is beneficial also for assessing the likelihood of patients' engagement in health-promoting behaviors. In fact, the specific behaviors that patient and partner demonstrate toward each other either increase or hinder illness management behaviors. For example, partners' active engagement coping style (i.e., discussing the situation with the patient, asking about feelings and constructive problem-solving activities; Coyne and Smith, 1994) was positively associated with patients' cessation of smoking, especially among those patients who perceived their partners' active engagement to be high (Vilchinsky et al., 2011). Moreover, partners' overprotective coping style (i.e., underestimation of patient's capabilities, offers of unnecessary aid or activity restrictions; Coyne and Smith, 1994) was associated with higher levels of patients' harmful blood lipids, especially among patients who perceived their partners' overprotection as high (Coyne and Smith, 1994).

## It most definitely takes two to cope with LVAD

Recent studies focusing on LVAD demonstrate how important it is do adopt the dyadic approach. Rossi Ferrario and Panzeri (2020) reported a worsening in the illness denial measure for both patients and caregiver and explored the caregiver's mourning (Rossi Ferrario et al., 2016). Later, Rossi Ferrario et al. (2022) found an associated level of anxiety, depression and strain among LVAD patients and their caregivers, Recently, Rapelli et al. (2023) used a dyadic phenomenological hermeneutic approach to explore the experiences of both patient and caregivers before discharging from the hospital into a new challenging home routine. Their findings shed light on important dyadic-related themes. For example, they detected the ambivalence around coping together, as patients were apprehensive about becoming a source of burden for their loved ones, while caregivers were concerned about experiencing increased stress due to the caregiving responsibilities. While Rapelli et al. acknowledged the importance of the dyadic approach, their data was collected individually (i.e., they interviewed the patient and the spouse separately. To advance the dyadic approach even further, we (Golan et al., 2023) conducted dyadic interviews (Eisikovits and Koren, 2010) and realized that the dyadic point of view is crucial for understanding patients and spouses emotional state and the nature and utility of their coping efforts.

What struck us as surprising was that in five interviews, although it was clear that the patients encountered difficulties, their individual attitude gave a misleading impression that they coped rather well. However, once their partners joined the conversation, the picture changed dramatically. The partners shared intensive feelings of anger, despair, and anxiety, which were expressed in an aggressive and blaming manner toward the patients (see quotations 1 and 2, Appendix A, taken from Golan et al., 2023). The partners' bursts of aggression toward their husbands were dramatic, authentic, and intense, and provided us with a glimpse of the harsh and toxic atmosphere in which these couples lived. It was clear that the patients experienced discomfort with their partners' aggression, but most of them did not respond. The patients' attitudes, which could have been mistakenly interpreted as adaptive if they had been interviewed alone, now appeared depressive. The dyadic setting allowed us to observe, in real time, how dyadic feelings were created, expressed, and responded to. This revealed the sad reality of these couples. What they were actually coping with was not the LVAD but rather the partners' aggression and anxiety and the patients' helplessness and depression.

We found an additional opportunity to observe real-time dyadic dynamics in quarrels triggered by the interview questions. For example, one couple argued whether it was worth having the operation. The patient described his helplessness and despair and even suicidal intention claiming that he regrets the decision to have the operation, while the spouse denied his feelings and could not provide any empathy (see quotation 3, Appendix A). Another spouse mocked her husband fear of being dependent upon a device (see quotation 4, Appendix A). These quarrels illustrate the partners' inability to provide their husbands with empathy and could not have been observed unless they had been interviewed together.

However, adopting the dyadic approach enabled us not only to reveal destructive coping dynamics, but also to observe efficient coping strategies that promoted couples' wellbeing. For example, some couples were highly synchronized, completing each other's sentences, as if they were reading each other's minds (see quotations 5 and 6, Appendix A). They way these couples responded represented a common narrative, identification, and support. An even deeper level of togetherness was expressed by some couples that refereed to their body perception almost as if they shared the same body (see quotations 7 and 8, Appendix A). This meaningful and rich data enabled us to have a much deeper understanding of the patients and spouses' experiences and could not have been obtained in individual interviews.

## Discussion

Previous research explored the importance of the dyadic approach in the context of LVAD with regards to rehabilitation programs (Rossi Ferrario et al., 2019), illness denial measure (Rossi Ferrario and Panzeri, 2020) and caregiver's mourning (Rossi Ferrario et al., 2016). We have demonstrated how the dyadic approach provides insights with regard to clinical outcomes, self-management of health-promoting behaviors, and emotional well-being in the context of cardiac illness and LVAD transplantation. Moreover, we illustrated how the dyadic interviewing methodology provided an opportunity to observe real-time authentic expressions of aggression and quarrels as well as support and identification. The dyadic interviewing allowed access to naturally occurring dynamics of couples' efforts to cope with the LVAD rather than retrospective, descriptive data that would likely have emerged in an individual interview with either patient or partner alone. Consequently, we argue that the dyadic point of view is a critical prism through which to explore coping with cardiac chronic illness. Yet, one important pitfall researchers should be aware of while adopting the dyadic approach is that participants may avoid saying content that they suspect would be offensive or embarrassing in front of their spouse. In such cases individual interview can be performed in addition to the dyadic one.

Given the importance and uniqueness of the data obtained, we conclude that in chronic disease situations characterized by a high level of dependence between patient and partner, applying an individual approach that does not grasp interpersonal interactions may lead to missing critical and valuable information and produce misleading conclusions. Therefore, researchers interested in studying patients' and caregivers' engagement in chronic disease management should apply a dyadic rather than individual approach, during both data collection and analysis. Only then can a fuller picture of the mechanisms leading to change and adjustment be revealed, and more efficient interventions developed. Our argument was vividly summarized by one of the nurses working in the LVAD clinic: “If I really want to know how he (the patient) is doing, all I need to do is ask his wife; sometimes even just looking at her is enough to figure out what's going on.”

## Author contributions

MG and NV contributed equally to writing, reviewing, reading, and approving the submitted version. This work was carried out at Bar-Ilan University as part of the first author's thesis under the supervision of the second author. Both authors contributed to the article and approved the submitted version.

## Appendix A

### Quotations demonstrating the importance of the dyadic approach taken from Golan et al. (2023)

Examples of anger, despair, and anxiety:

1. “Since the operation, I am a slave. It's really impressive that the doctors saved his life (pointing at her husband without looking at him), but did they think of me? (shouting) I am almost 80, why did no one ask me in advance if I am capable of doing this? I can't take it any longer (banging on the table with her hands).”
2. “This is very difficult, physically and emotionally. I am the driver, the eyes, the doctor. I had to give up all the joy and fun I had in my life (looking at her husband with a blaming, angry look).”
3. Patient: “You have a hole in your body. You can't take a shower. This is terrible!” Partner (interrupting him): “But you are alive!” Patient (yelling): “But I can't walk! I wouldn't even mind if the batteries stopped working. I will not hurry to replace them.” Partner: “But you are alive! You can breathe.”
4. Patient: “This is very scary. I am dependent upon a device. If it stops, you just die.” Partner: “Really!!(giggling) Of all things, this is not so scary.”

Examples of efficient coping strategies that promoted couples' wellbeing:

1. Patient: “I was in a very bad condition. I practically died. My heart stopped beating for…”(looks at his partner to recall). Partner: “For seven whole minutes!”
2. Patient: “I found myself on the floor and my whole body was covered with blood.” Partner: “His whole body was covered with blood, the whole floor with blood.” Patient: “The whole living room was full of blood, and I couldn't call her for help because I lost my voice.” Partner: “He couldn't call me. He lost his voice.”
3. Patient: “I started to feel unusual pain, strong pains.” Interviewer: “What kind of pain did you feel?” Partner: “It was in his leg, where he usually doesn't have any pain.”
4. Partner: “We need to charge the batteries. This is truly scary. If we fail to do it, it's dangerous.” Patient: “Indeed, it is scary.” Partner: “We feel the fear together. We are one body. What he goes through, I go through.”
